# A simple and available measurement of onco-sEV dsDNA to protein ratio as a potential tumor marker

**DOI:** 10.1186/s12885-023-10886-3

**Published:** 2023-07-03

**Authors:** Yifan Sun, Miao Li, Xiaoshan Zhang, Dongjie Xu, Jie Wu, Xinrui Gu, Adeel Khan, Han Shen, Zhiyang Li

**Affiliations:** 1 Department of Laboratory Medicine, Nanjing Drum Tower Hospital Clinical College of Jiangsu University, Nanjing, China; 2 Department of Laboratory Medicine, Nanjing Drum Tower Hospital, The Affiliated Hospital of Nanjing University Medical School, Nanjing, China; 3 College of Life Science, Yangtze University, Jingzhou, China; 4 State Key Laboratory of Bioelectronics, School of Biological Science and Medical Engineering, National Demonstration Center for Experimental Biomedical Engineering Education (Southeast University, Southeast University, Nanjing, China

**Keywords:** Small extracellular vesicles, Double-stranded DNA, Nucleic acid to protein ratio, Tumor diagnosis

## Abstract

**Background:**

Small extracellular vesicles (sEVs) have great potential as new biomarkers in liquid biopsy. However, due to the limitations of sEVs extraction and component analysis procedures, further clinical applications of sEVs are hampered. Carcinoembryonic antigen (CEA) is a commonly used broad-spectrum tumor marker that is strongly expressed in a variety of malignancies.

**Results:**

In this study, CEA^+^ sEVs were directly separated from serum using immunomagnetic beads, and the nucleic acid to protein ultraviolet absorption ratio (NPr) of CEA^+^ sEVs was determined. It was found that the NPr of CEA^+^ sEVs in tumor group was higher than that of healthy group. We further analyzed the sEV-derived nucleic acid components using fluorescent staining and found that the concentration ratio of double-stranded DNA to protein (dsDPr) in CEA^+^ sEVs was also significantly different between the two groups, with a sensitivity of 100% and a specificity of 41.67% for the diagnosis of pan-cancer. The AUC of dsDPr combined with NPr was 0.87 and the ACU of dsDPr combined with CA242 could reach 0.94, showing good diagnostic performance for pan-cancer.

**Conclusions:**

This study demonstrates that the dsDPr of CEA^+^ sEVs can effectively distinguish sEVs derived from tumor patients and healthy individuals, which can be employed as a simple and cost-effective non-invasive screening technology to assist tumor diagnosis.

**Supplementary Information:**

The online version contains supplementary material available at 10.1186/s12885-023-10886-3.

## Introduction

Small extracellular vesicles (sEVs) are lipid bilayer membrane vesicles with a diameter of 30–150 nm. In recent years, sEVs have shown tremendous promise in liquid biopsy due to their good stability, high abundance in body fluids, and capacity to transport genetic information from parental cells [1–3]. The examination of bioinformatic components carried by circulating tumor sEVs, such as nucleic acids and proteins, can provide effective information for cancer diagnosis, monitoring and prognosis prediction. At present, a variety of methods have been established for isolation and detection of sEVs content [4–10], but there are still issues that prevent sEVs from being used clinically, including the complicated isolation of sEVs and the absence of tumor-specific markers [11–14].

Studies have reported that double-stranded DNA (dsDNA) is the predominant form of sEV-derived DNA and is primarily encapsulated within larger sEVs (80–200 nm)[15, 16]. Recently, sEV-derived dsDNA has attracted attention as a biomarker for cancer diagnosis with multiple detection methods, including flow cytometry, real-time PCR, digital PCR, next-generation sequencing, etc. [17–19]. In this study, we detected the nucleic acid to protein ratio (NPr) of CEA^+^ sEVs by standard UV spectroscopy and determined the concentration ratio of dsDNA to protein (dsDPr) of CEA^+^ sEVs by fluorescent staining, to distinguish sEVs derived from tumor patients and healthy individuals. The findings indicated that the method analyzes sEVs as a whole object, which does not require the extraction of serum sEVs and is not restricted to the analysis of a single or a class of indicators, establishing a simple and economical method for tumor diagnosis.

## Materials and methods

### Research object

In this study, 34 tumor patients treated in Nanjing Drum Tower Hospital from May 2021 to September 2021 were selected. The average age of tumor group was 59 years old, with 25 male and 9 female. There were 13 cancer types in cancer group, including gastric cancer, lung cancer, colorectal cancer and so on. The average age of healthy group was 49 years old, with 18 male and 16 female. Details of the study subjects are provided in Supplementary Tables 1–2. This study was approved by the Ethics Committee of Nanjing Drum Tower Hospital. All methods were performed in accordance with the relevant guidelines and regulations.

### Cell culture

The cell lines A549, Beas-2b, SGC7901 and GES cells were purchased from Cobioer bioscience (Nanjing, China). A549 and Beas-2b cells were cultured in DMEM medium (Gibco, USA) containing 10% fetal bovine serum (FBS), while SGC7901 and GES were cultured in RPMI-1640 medium (Gibco, USA) with 10% FBS. All cells were maintained in a humidified incubator (Thermofisher, SHKE8000-8CE) at 37℃ with 5% CO_2_.

### sEVs isolation from cells

The cells were cultured with correspond media contain 10% sEV-free FBS medium for 24 h, when cell growth density reaches 50–60% in common media. Then the medium was collected and centrifuged at 300 g for 10 min, followed by 2000 g for 30 min to remove cell debris. After that the supernatant was filtered using a 0.22 μm pore filter (Merck Millipore, German) to remove the apoptotic bodies, shedded vesicles and cell debris. The collected filtered supernatant was centrifuged at 100,000 g for 70 min at 4°C (Beckman Coulter Optima XE-100, Type 90 Ti rotor) twice to collect sEVs. The pelleted sEVs were resuspended in 100 µL phosphate-buffered saline (PBS).

### sEVs isolation from human serum samples

The collected serum was centrifuged at 300 g for 10 min and 2000 g for 30 min at 4 °C to remove the cell and cell debris before storing at -80°C. For the isolation of sEVs from serum, the frozen serum was thawed at 4°C and centrifuged at 10,000 g for 10 min at 4°C. After that, the supernatant was filtered through a 0.22 μm pore filter to remove residual contamination. Then, the supernatant was centrifuged at 100,000 g for 70 min at 4°C twice to collect sEVs, the pellets resuspended in 100 µL phosphate-buffered saline (PBS).

### Nanoparticle tracking analysis (NTA)

The concentration and size distribution of sEVs were measured using Zetaview (German, PMX120). The samples were diluted 1000-fold with PBS for detection. Each sample was diluted in triplicate and each diluted sample was analyzed 3 times using the same settings for detection.

### Transmission Electron Microscopy

The cell-derived sEVs (5⋅10^9^/mL) were used for transmission electron microscopy (TEM) to examine the morphology and size of sEVs. The sEVs sample was deposited onto the ultrathin copper mesh. The TEM experiments were performed at the Analysis Center of Southeast University.

### Quantitative real-time PCR (qRT-PCR)

Total cellular RNA was extracted from cells with TRIzol reagent (Invitrogen, USA) and then subjected to reverse transcription using a PrimeScript™ RT Master Mix Kit. (Takara, Japan). qRT-PCR was performed on a Step One Plus™ PCR system (Applied Biosystems, 7500) using ChamQ™ SYBR® qPCR Master Mix (Vazyme, China), follow the instructions of the guidance. The primer sequences were as follows: CEA, forward 5′- GCACCTCAGACCAATCATCAACT-3′, reverse 5′- CCACTTCTCAAGGACCAAATACAC-3′; GAPDH, forward 5′-GAAGGTGAAGGTCGGAGTCA-3′, reverse 5′-TTGAGGTC AATGAAGGGGTC-3′.

### Western blot

Cells were lysed in RIPA buffer containing 1mM PMSF and the protein concentration was quantified using the BCA protein assay reagent kit. The samples were separated by SDS-PAGE and transferred to PVDF membrane. Then, the membrane was blocked with 5% non-fat milk and subsequently incubated with primary antibody as follows: CD63 (ab134045, Abcam), TSG101 (ab125011, Abcam), Calnexin (ab133615, Abcam) and GAPDH (10494-1-AP, Proteintech), characteristic proteins that have previously been reported to be expressed on the surface of sEVs[20]. Afterwards, the membrane was incubated with the HRP conjugated secondary antibody at room temperature for 1 h. The bands were detected by the ultra-ECL regent kit (Beyotime Shanghai, China).

### Nucleic acid to protein ratio (NPr) measurement

Since nucleic acid and protein have the maximum absorption peaks at 260 and 280 nm, respectively, the maximum 260-280 nm absorbance ratio of sEVs was analyzed to assess the nucleic acid and protein ratio of sEVs which called NPr. To detect the NPr of cell and serum-derived sEVs, 1.5 µL sEVs were directly aspirated to determine the ratio of absorbance at 260 to 280 nm by a UV absorption spectrophotometer (Nanodrop 2000, USA). For the detection of CEA^+^ sEVs, CEA^+^ sEVs were isolated from serum using magnetic bead modify with CEA antibody (Siemens, USA) to detect their NPr. Briefly, 100 µL of serum was mixed with 250 µL of magnetic bead modify with CEA antibody in a 1.5 mL tube and incubated on a rotator for 30 min at room temperature. The tubes were placed in a magnetic field and the supernatants were removed after standing for 1 min. Then, the magnetic beads were washed 3 times with 200 µL of PBST, dissolved in 20 µL of DEPC water, and heated at 95 °C for 10 min to dissociate nucleic acids and proteins from the magnetic bead. The supernatant was separate by magnetic rack. Finally, as described above, 1.5 µL of supernatant was taken to detect the NPr of CEA^+^ sEVs.

### Double-stranded DNA to protein ratio (dsDPr) measurement

The isolation of CEA^+^ sEVs followed the NPr assay procedure. The absolute quantitative of dsDNA and protein were carried out according to the product instructions (Thermofisher, USA). Specifically, 1 µL of CEA^+^ sEVs lysis was combined with 199 µL of working solution, then vortexed and incubated for 2 min (dsDNA) or 15 min (proteins) at room temperature. The Qubit Flex Fluorometer was used to quantify the sample concentration.

### Statistical analysis

All calculations were performed by GraphPad Prism 9. Statistical analyses were performed using Student’s t-test, ANOVA and Fisher’s extract test. P < 0.05 was considered statistically significant. The results are expressed as the mean ± SEM for three independent experiments.

## Results and discussion

### Scheme of serum SEVs detection

**Fig. 1:**
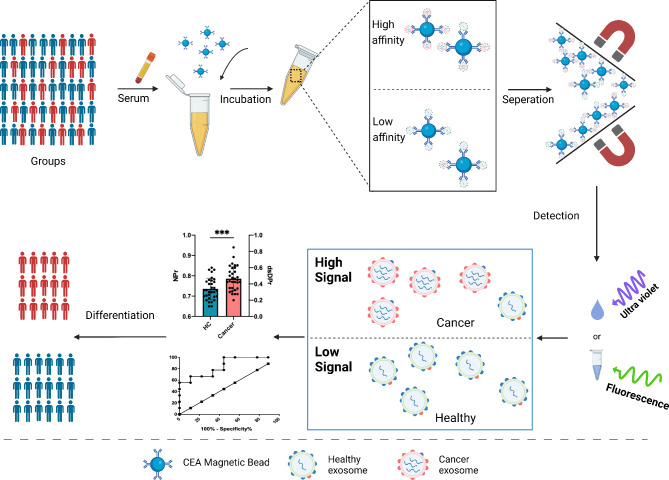
**Scheme of serum sEVs detection** The detection scheme of NPr and dsDPr was presented in Fig. 1. Serum from healthy individuals and patients with different types of tumors were collected. CEA^+^ sEVs were enriched using CEA magnetic bead antibody. The absorbance ratio of CEA^+^ sEVs at A260 and A280 (NPr) was compared using UV spectrophotometry, and the concentration ratio of dsDNA and protein (dsDPr) in CEA^+^ sEVs was detected by fluorescent dye method

The detection scheme of NPr and dsDPr was presented in Fig. 1. Firstly, serum from healthy individuals and patients with different types of tumors were collected. Then, CEA^+^ sEVs were enriched from a large number of non-tumors sEVs using CEA magnetic bead antibody to lessen the signal interference caused by non-tumor sEVs. After the incubation, the magnetic beads and sEVs were dissociated by heating, and the supernatant was separate by magnetic rack for subsequent detection. The absorbance ratio of CEA^+^ sEVs at A260 to A280 was compared using UV spectrophotometry, and the concentration ratio of dsDNA and protein in CEA^+^ sEVs was detected by fluorescent dye method, which could effectively distinguish tumor and non-tumor derived sEVs. Thus, a simple and economical method for tumor auxiliary diagnostic was established.

### NPr of Tumor Cell sEVs is higher than Non-Tumor Cell sEVs

**Fig. 2:**
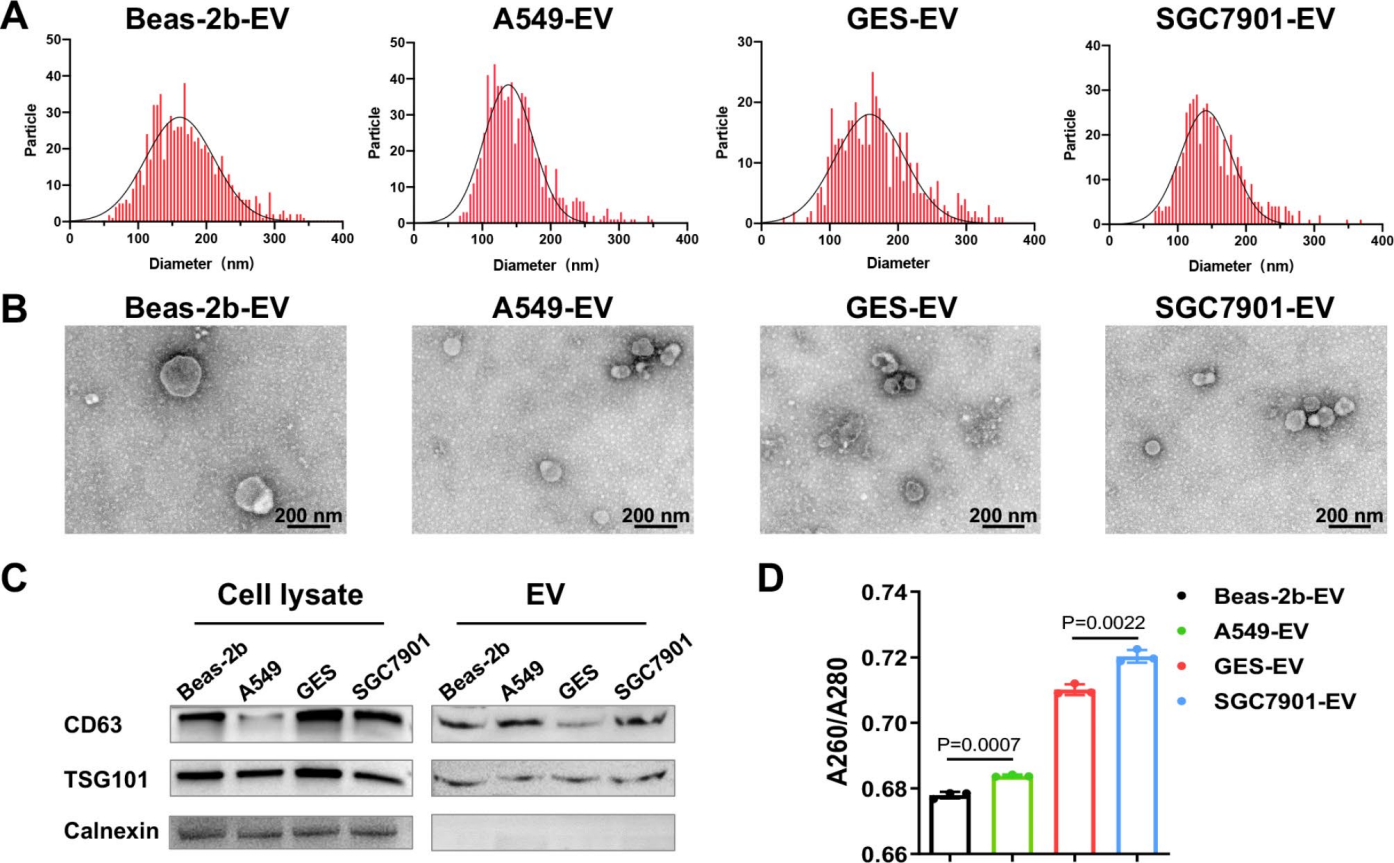
**Characterization and NPr of tumor cell and non-tumor cell sEVs** (A) Particle size distributions of the four cellular sEVs. (B) TEM of the four cellular sEVs. (C) Protein expressions of cell lysates and sEVs. (D) NPr of tumor cell and non-tumor cell sEVs.

Tumor sEVs have been found to contain more DNA than sEVs originating from non-tumor cells. Due to the variation in molecular structure, the maximal UV absorption peak of nucleic acid is at 260 nm, while that of protein is at 280 nm. Thus, the absorbance ratio of sEVs at 260 and 280 nm can be used to evaluate their relative nucleic acid to protein ratio (NPr). We extracted sEVs from four cell lines, including two tumor cells (A549 and SGC7901) and two non-tumor cells (Beas-2b and GES). The characterization results showed that the particle size distributions of the four sEVs ranged from 50 to 200 nm, which were disc-shaped vesicles (Fig. 2A-B). They expressed the sEV positive proteins CD63 and TSG101, but not the negative protein Calnexin (Fig. 2C). Further, the NPr of sEVs in each group were detected, and it was found that the NPr values of sEVs derived from lung cancer cells and gastric cancer cells were higher than their corresponding control groups (Fig. 2D), implying the potential of NPr to distinguish tumor and non-tumor derived sEVs.

### NPr of CEA^+^ sEVs can differentiate sEVs from Tumor Patients and healthy individuals

**Fig. 3:**
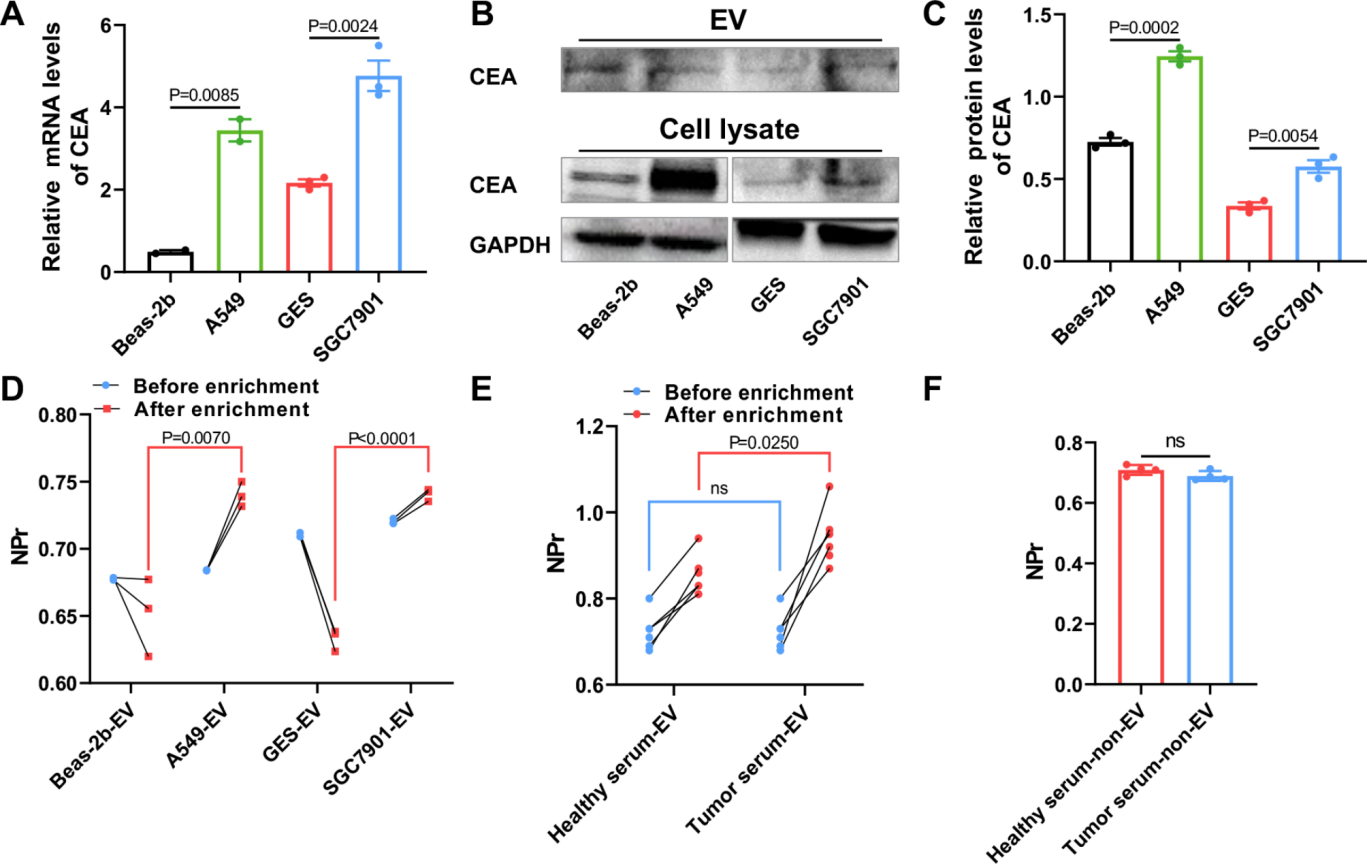
**CEA magnetic bead antibody can effectively capture CEA** ^**+**^ **sEVs in cell supernatant and serum** (A) mRNA expression of CEA in the four cell lines. (B) Protein expression of CEA in the cell lysates and sEVs. (C) Statistical analysis of CEA protein expression. (D) NPr of cell sEVs before and after enrichment of CEA magnetic bead antibodies. (E) NPr of serum sEVs before and after enrichment of CEA magnetic bead antibodies. (F) NPr of serum without sEVs.

Unlike cell line-derived sEVs, the source of sEVs in serum is plentiful, and tumor sEVs only account for a minor portion of them. We used immunomagnetic beads to enrich the sEVs expressing CEA, a tumor marker that is highly expressed in various tumor cells, to reduce the interference produced by non-tumor cell sEVs by detecting the NPr of CEA^+^ sEVs. The results showed that CEA was highly expressed in A549 and SGC7901 cells, as well as their sEVs (Fig. 3A-C). Additionally, the NPr in both tumor groups are significantly increased after enrichment, indicating that the magnetic bead antibody can effectively enrich CEA^+^ sEVs in cellular sEVs (Fig. 3D). Then, the feasibility of the method was validated in clinical serum samples. The findings revealed that the NPr of CEA^+^ sEVs in the serum of tumor patients was significantly higher than that of the healthy control group (Fig. 3E). However, there was no considerable difference between the two groups when the sEVs in serum were removed (Fig. 3F). The above results indicated that the CEA magnetic bead antibody can effectively enrich CEA^+^ sEVs in patient serum, and the NPr of CEA^+^ sEVs can distinguish tumor patients and non-tumor patient-derived sEVs.

### Interference experiments of CEA^+^ sEV NPr

**Fig. 4:**
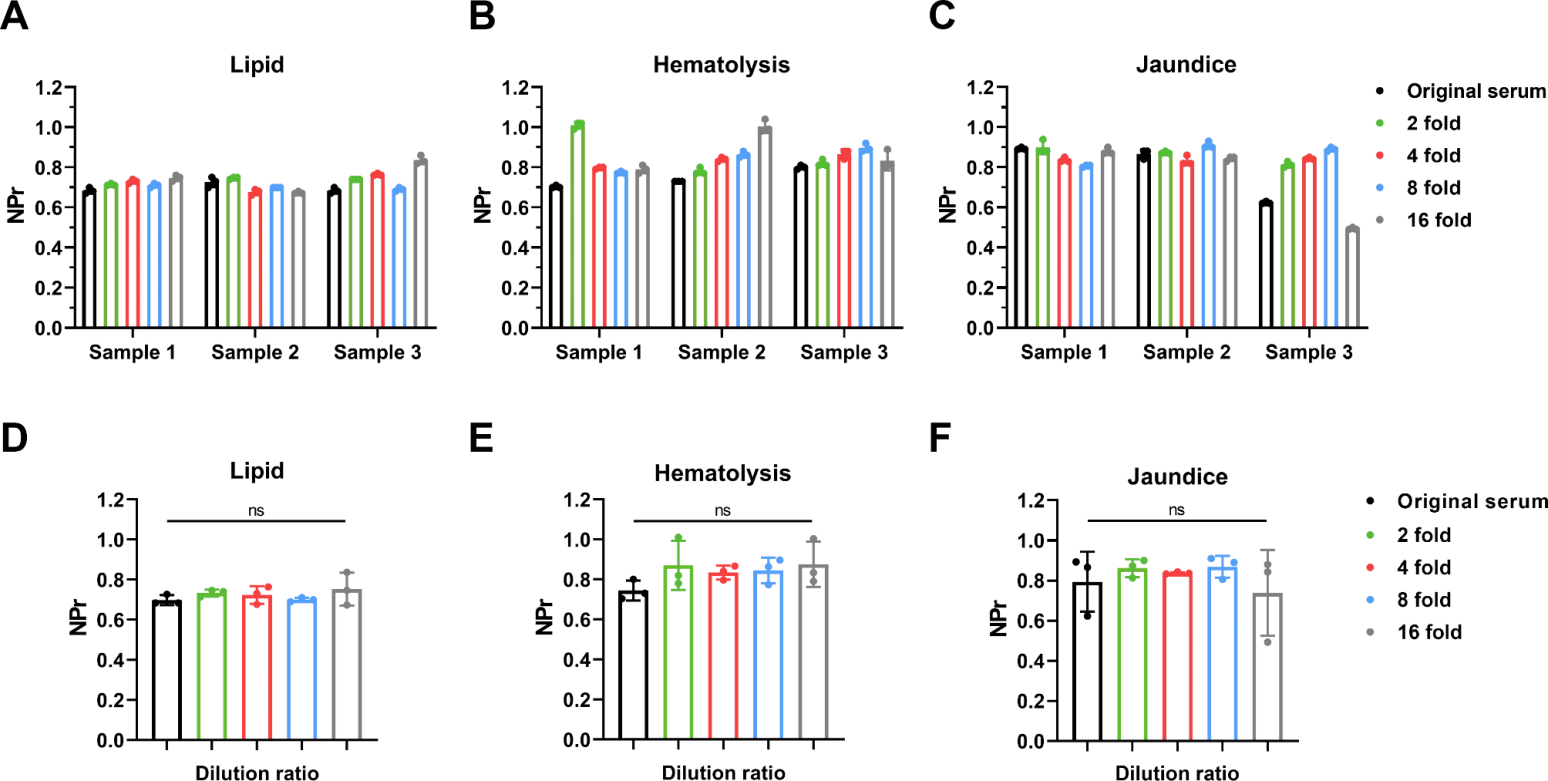
**Interference experiments of CEA** ^**+**^ **sEV NPr** (A) NPr of CEA^+^ sEV with lipid at different dilution concentrations. (B) NPr of CEA^+^ sEV with hemolysis at different dilution concentrations. (C) NPr of CEA^+^ sEV with jaundice at different dilution concentrations. D-F. Statistical analyses of NPr of CEA^+^ sEV with lipid, hemolysis and jaundice

We further assessed the anti-interference capability of the NPr detection method in intricate clinical specimens in light of the practical clinical use. The blood samples with varying degrees of lipid, hemolysis or jaundice were collected and performed NPr detection after dilution, which was a common method to reduce background noise in practical work (Fig. 4A-C). The findings demonstrated that the NPr obtained by this approach can maintain relatively stable results in the presence of lipid, icterus or hemolysis interference, and there were no statistically significant differences between the samples diluted by various rates (Fig. 4D-F).

### dsDPr of CEA^+^ sEVs can be used for Tumor Adjuvant diagnosis

**Fig. 5:**
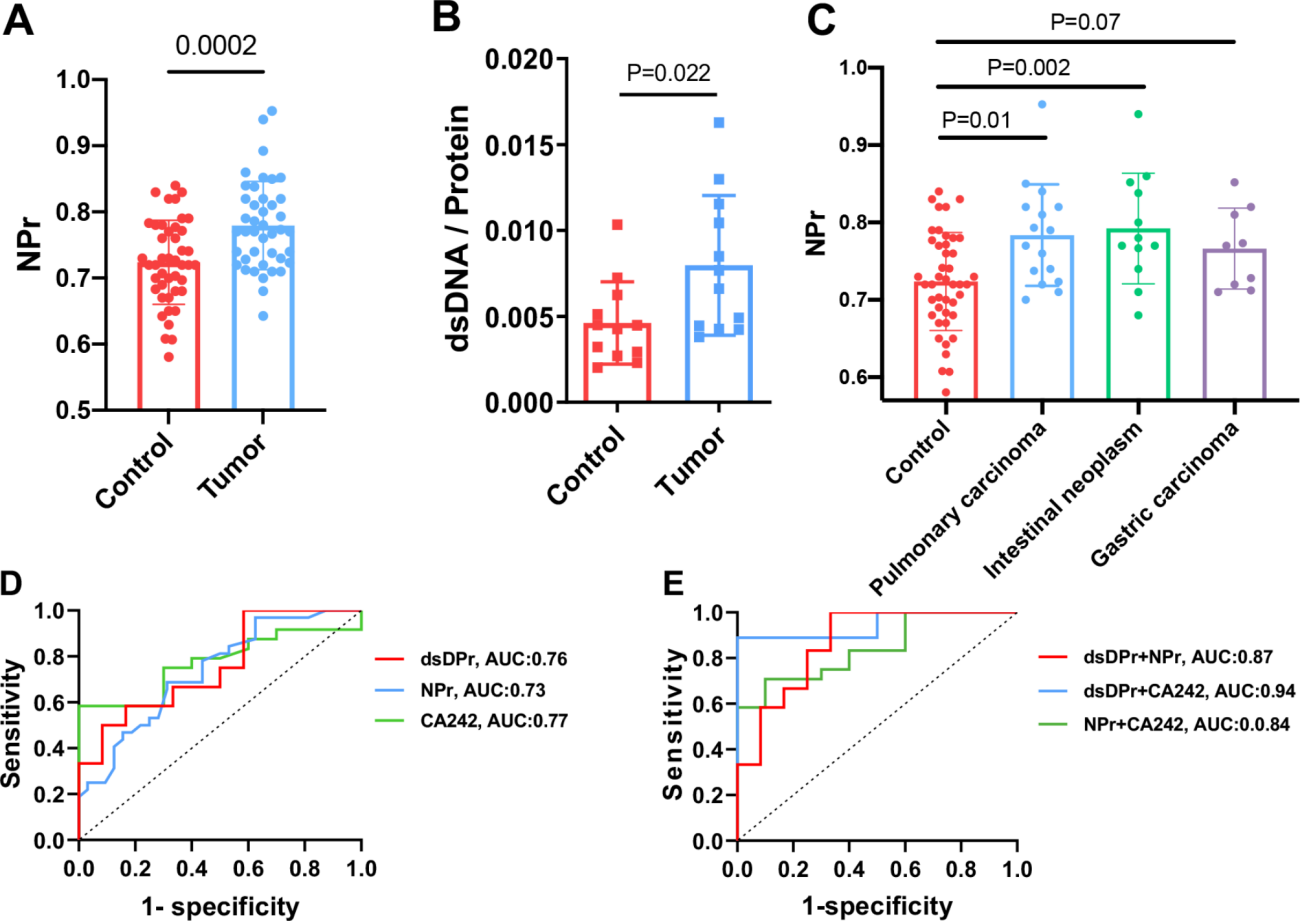
**The role of NPr and dsDPr of CEA** ^**+**^ **sEVs in the auxiliary diagnosis of tumors** (A) NPr of CEA^+^ sEVs from tumor patients and healthy individuals. (B) dsDPr of CEA^+^ sEVs from tumor patients and healthy individuals. (C) NPr of CEA^+^ sEVs from different tumor source patients and healthy individuals (Control n = 44, Pulmonary carcinoma n = 16, Intestinal neoplasm n = 12, Gastric carcinoma n = 9). D-E. ROC curve analysis of various indicators

To verify the difference of CEA^+^ sEVs NPr in serum, we examined serum samples from patients with different types of tumors and found that the NPr of CEA^+^ sEVs in the tumor group was significantly higher than that in the healthy control group (n = 44) (Fig. 5A). The sEVs have been reported to transport a range of nucleic acids, including DNA, RNA, miRNA, lncRNA, etc. The expressions of dsDNA, RNA and miRNA in CEA^+^ sEVs were further detected using fluorescent staining, and it was found that the ratio of dsDNA to protein (dsDPr) in the tumor group was significantly higher than that in the control group (Fig. 5B), implying that the difference in NPr between the two groups could be due to the dsDNA in sEVs. Furthermore, we grouped samples by tumor source to assess their predictive power for specific tumors. It can be seen from the (Fig. 5C) that CEA^ + ^sEVs NPr has excellent diagnostic ability for pulmonary carcinoma and intestinal neoplasm, and the reason for its slightly poor diagnostic effectiveness for gastric cancer may be because the number of gastric cancer samples is still insufficient. As shown in Supplementary Fig. 1, both NPr and dsDPr followed normal distributions. In terms of tumor diagnosis, the sensitivity and specificity of CEA^+^ sEVs NPr were 68.75%, and the sensitivity and specificity of dsDPr were 100% and 41.67%, respectively. The results of ROC curve analysis showed that the AUCs of NPr, dsDPr and their combination in the diagnosis of pan-cancer were 0.73, 0.76 and 0.87, respectively. In addition, CA242 is a tumor marker with high specificity, and its combined diagnostic efficiency with dsDPr can reach 0.94, showing good diagnostic value for pan-cancer. The above results indicated that the dsDPr of CEA^+^ sEVs can be used as potential markers for auxiliary diagnosis of tumors (Fig. 5D-E).

## Discussion

Liquid biopsy has the advantages of less invasiveness, convenient sample collection, and dynamic monitoring. Circulating tumor cells (CTCs), circulating tumor DNA (ctDNA) and sEVs are known as the “troika” of liquid biopsy. Compared with CTCs and ctDNA, sEVs have been shown to have greater application prospects in the early diagnosis, monitoring and prognosis prediction of tumors [14, 15, 21–23]. Studies reported that exosomal proteins found in patient serum and urine, such as LRP1, EGFR and LG3BP, have been associated to lung cancer stage and metastasis [24–28]. In HCC patients, the exosomal miR-92b could predict early recurrence of HCC with an AUC of 0.925, which was better than circulating AFP with an AUC of 0.651[29]. Li et al. demonstrated that tRNA-GlyGCC-5 and a previously undocumented small RNA were shown to be preferentially concentrated in salivary sEVs of ESCC patients and the bi-signature consisting of these small RNAs was able to discriminate ESCC patients from the controls with high sensitivity (90.50%) and specificity (94.20%) [30]. It has also been reported that DNA in circulating sEVs also plays a role in the occurrence and development of diseases[31]. It is encouraging that sEV-based liquid biopsies have been tested in clinical trials and some of them have been approved for the market. For example, ExoDx™ Prostate IntelliScore™ is the first sEV-based liquid biopsy product to receive breakthrough medical device identification, which assesses prostate cancer aggressiveness by analyzing three biomarkers found in exosomal RNA in urine. Despite significant advancements, sEVs detection still confronts numerous hurdles, including complicated sEVs extraction, lack of specific tumor markers, and presence of sEVs heterogeneity.

Tumor antigens and ectopic hormones, such as CEA, AFP, NSE, CA50, CA724, CA125, etc., are currently the most often utilized tumor biomarkers in clinic [32–36]. CEA overexpress in several type of tumor which associate with cell proliferation and tumor progression is a broad-spectrum tumor marker that can be used to assess efficacy, monitor disease progression, and predict prognosis in lung cancer, colorectal cancer, breast cancer, and other cancers [37–40]. We used the CEA antibody magnetic bead to directly enrich the sEVs carried CEA in serum and detected the NPr of the CEA^+^ sEVs through UV spectroscopy. The results revealed that the NPr of the tumor group was significantly higher than that of the control group, indicating the effectiveness of our approach in differentiating tumor-derived sEVs from non-tumor-derived sEVs. In addition, the stability of the detection results of traditional tumor markers is often affected by degradation and interference of substances in the blood. We further found that in the presence of lipid, hemolysis and jaundice interferences, the detection of NPr was almost unaffected, which was more stable than the traditional quantitative detection of single protein or nucleic acid markers. Proteins or nucleic acids in the blood are prone to degradation by the action of various enzymes. Hemolysis, lipids and jaundice can also affect the detection results of some tumor markers, such as hemolysis can lead to elevated results of ferritin. Nucleic acids and proteins in sEVs are protected by lipid layers and not susceptible to degradation, and NPr detects sEVs as a whole, rather than a single protein or nucleic acid alone, thus reducing the effects of distractors and degradation. In line with our findings, D. Sun et al. also demonstrated that tumor and non-tumor derived sEVs can be distinguished by detecting the NPr of sEVs [41]. However, the sEVs needed to be extracted from cells and serum before detection, which is not suitable for clinical testing. Compared to some commonly used clinical detection methods, such as enzyme-based sEVs detection method, fluorescence detection method, and electrochemical detection method, our method does not require special equipment and is simple to operate. sEVs carry a variety of bioactive molecules including nucleic acids, proteins and lipids, which can be transferred from parental cells to recipient cells [42–45]. In this study, apart from the difference in NPr, we analyzed the nucleic acid components in CEA^+^ sEVs, including dsDNA, RNA and miRNA, and found that the ratio of dsDNA to protein in the tumor group was also significantly higher than that in the healthy group. Moreover, dsDPr displayed comparable diagnostic efficacy to CA242 which play important role in pancreatic cancer[46] in the diagnosis of pan-cancer and had higher tumor diagnostic efficiency when used in combination with CA242.

Overall, our results provided evidence for dsDPr of CEA^+^ sEVs as a potential marker for pan-cancer diagnosis with a simple and economical assay that can be applied to tumor screening. However, the specificity of our method is not enough. It is necessary to graft detection methods or indicators with higher specificity, so as to be applied to the accurate diagnosis of tumors. For example, dsDPr can be used in combination with traditional tumor markers (such as CA242) to improve the accuracy of tumor diagnosis. Han et al. developed a method for the programmable autonomous synthesis of DNA based on the Primer exchange reaction [47], which we can introduce to improve the sensitivity and specificity of our detection method.

## Conclusions

In brief, this study demonstrated that the dsDPr of CEA^+^ sEVs can effectively distinguish sEVs derived from tumor patients and healthy individuals, which can be employed as a simple and cost-effective non-invasive screening technology to assist tumor diagnosis.

## Electronic supplementary material

Below is the link to the electronic supplementary material.

Supplementary Material 1: Tables 1 and (2) Details of the study subjects. Figure 1. Normal distribution of NPr and dsDPr.

## Data Availability

The data sets generated or analyzed during this study are available from the corresponding author on reasonable request.
