# Supplementary material for: A simple and available measurement of onco-sEV dsDNA to protein ratio as a potential tumor marker
Source: BMC Cancer. 2023 Jul 3;23:614. doi: 10.1186/s12885-023-10886-3 (PMC10316596; doi:10.1186/s12885-023-10886-3)
Supplement: Supplementary file 1 — Supplementary Material 1: Tables 1 and (2) Details of the study subjects. Figure 1. Normal distribution of NPr and dsDPr. [file 12885_2023_10886_MOESM1_ESM.docx]

Supplementary Table1.The information of health donors

| Serial Number | Gender | Age |
| --- | --- | --- |
| 1 | Male | 58 |
| 2 | Male | 29 |
| 3 | Female | 38 |
| 4 | Male | 32 |
| 5 | Male | 38 |
| 6 | Female | 33 |
| 7 | Male | 69 |
| 8 | Male | 38 |
| 9 | Male | 51 |
| 10 | Male | 54 |
| 11 | Female | 59 |
| 12 | Male | 58 |
| 13 | Female | 56 |
| 14 | Female | 55 |
| 15 | Male | 66 |
| 16 | Male | 62 |
| 17 | Male | 58 |
| 18 | Male | 27 |
| 19 | Female | 77 |
| 20 | Female | 56 |
| 21 | Male | 57 |
| 22 | Female | 76 |
| 23 | Male | 29 |
| 24 | Female | 78 |
| 25 | Male | 59 |
| 26 | Male | 47 |
| 27 | Male | 37 |
| 28 | Female | 54 |
| 29 | Female | 43 |
| 30 | Female | 28 |
| 31 | Female | 38 |
| 32 | Female | 29 |
| 33 | Female | 31 |
| 34 | Female | 54 |
| 35 | Male | 58 |
| 36 | Male | 37 |
| 37 | Female | 54 |
| 38 | Female | 45 |
| 39 | Male | 34 |
| 40 | Female | 27 |
| 41 | Male | 71 |
| 42 | Male | 54 |
| 43 | Female | 32 |
| 44 | Female | 43 |

Supplementary Table2. The information of cancer patients

| Serial Number | Gender | Age | Histology |
| --- | --- | --- | --- |
| 1 | Male | 62 | Right-sided lung cancer with extensive metastasis in the lung and lymphatic vessels |
| 2 | Female | 50 | Descending colon cancer (postoperative stage I IV (liver and lung)) |
| 3 | Male | 68 | Hypofractionated adenocarcinoma of the liver |
| 4 | Male | 58 | Lung cancer stage IV (meningeal) |
| 5 | Male | 71 | Tubular adenocarcinoma, IIIA (T3, N2, cM0) |
| 6 | Male | 63 | Adenocarcinoma stage IA |
| 7 | Male | 49 | Lung adenocarcinoma, chemotherapy |
| 8 | Male | 73 | Hepatocellular carcinoma stage IV A (T3, N1b, M1a), chemotherapy |
| 9 | Male | 64 | Stage IV pancreatic cancer (abdominal), chemotherapy |
| 10 | Male | 44 | Targeted therapy for malignant tumors |
| 11 | Male | 49 | Thyroid Cancer |
| 12 | Male | 33 | Gastric cancer stage IV |
| 13 | Male | 44 | Multiple polyps in the stomach |
| 14 | Female | 46 | Adenoma of the colon |
| 15 | Male | 57 | Colonic polyps |
| 16 | Female | 68 | Rectal cancer stage I (T1,N0,cM0) |
| 17 | Male | 75 | Stage IV hypofractionated adenocarcinoma of the right lung (T4N3M1b1 bone) |
| 18 | Male | 71 | Cardia gastric fundic malignancy (intermediate differentiated adenocarcinoma IIIB (T3N1cM0))，postoperative chemotherapy |
| 19 | Female | 60 | Small curvilinear adenocarcinoma of the cardia, IIIB (T4a,N3a,cM0) |
| 20 | Male | 74 | Stage IV adenocarcinoma of the right lung (pleura, bone, adrenal gland) |
| 21 | Female | 68 | Rectal cancer stage IV (liver, abdomen) |
| 22 | Male | 70 | Stomach cancer (post-operative) |
| 23 | Male | 70 | Right lung squamous carcinoma (stage IV cT2N2M1b adrenal) |
| 24 | Female | 32 | Gastric cancer stage IV (pelvic) |
| 25 | Male | 65 | Malignant tumor of colon (recurrent stage IV post- operative colon cancer (liver, lung)) |
| 26 | Female | 63 | Malignant tumor of colon (stage IV, liver metastasis) |
| 27 | Male | 55 | Intermediate differentiated adenocarcinoma of the rectum stage IV (both lungs, peritoneum) |
| 28 | Male | 68 | Sigmoid colon cancer (T3N2aM1 stage IV (liver metastasis)) |
| 29 | Male | 68 | Stage IV sigmoid colon cancer (liver) |
| 30 | Male | 68 | Stage IV adenocarcinoma of right lung (lung, bone, lymph node, soft tissue) |
| 31 | Male | 65 | Malignant tumor of colon (postoperative recurrence of colon cancer stage IV (liver and lung)) |
| 32 | Female | 59 | Malignant tumor of right lung (adenocarcinoma stage IV, cT2N3M1c) |
| 33 | Female | 32 | Gastric cancer stage IV (pelvic cavity) |
| 34 | Female | 55 | Malignant tumor of gallbladder (stage IV) |
| 35 | Male | 75 | Malignant tumor of colon (stage IV liver metastasis) |
| 36 | Male | 57 | Stage IV small cell carcinoma of the left lung |
| 37 | Female | 75 | Pancreatic tumors |
| 38 | Female | 55 | gastric cancer |
| 39 | Male | 74 | Stage IV gastric cancer |
| 40 | Female | 52 | Stage IV adenocarcinoma of the left lung |
| 41 | Male | 51 | Squamous cell carcinoma of the right lung |
| 42 | Male | 59 | Pulmonary mass |

Supplementary Figure1. Normal distribution of NPr and dsDPr.

Fig 2 C CD63


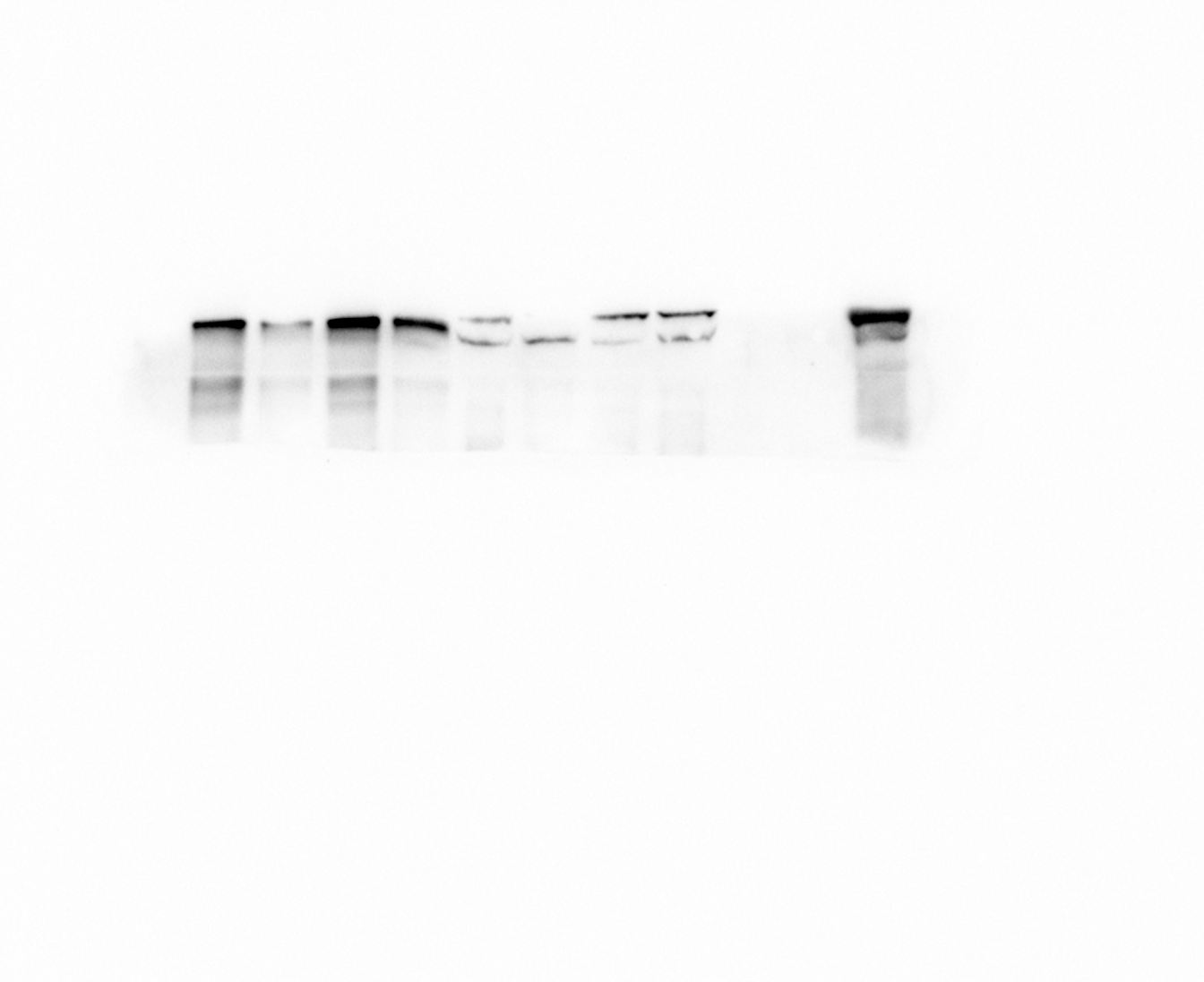


10s

**A549**

**Beas-2b**

**sEV**

**GES**

**SGC7901**

**Cell lysate**

**A549**

**Beas-2b**

**GES**

**SGC7901**


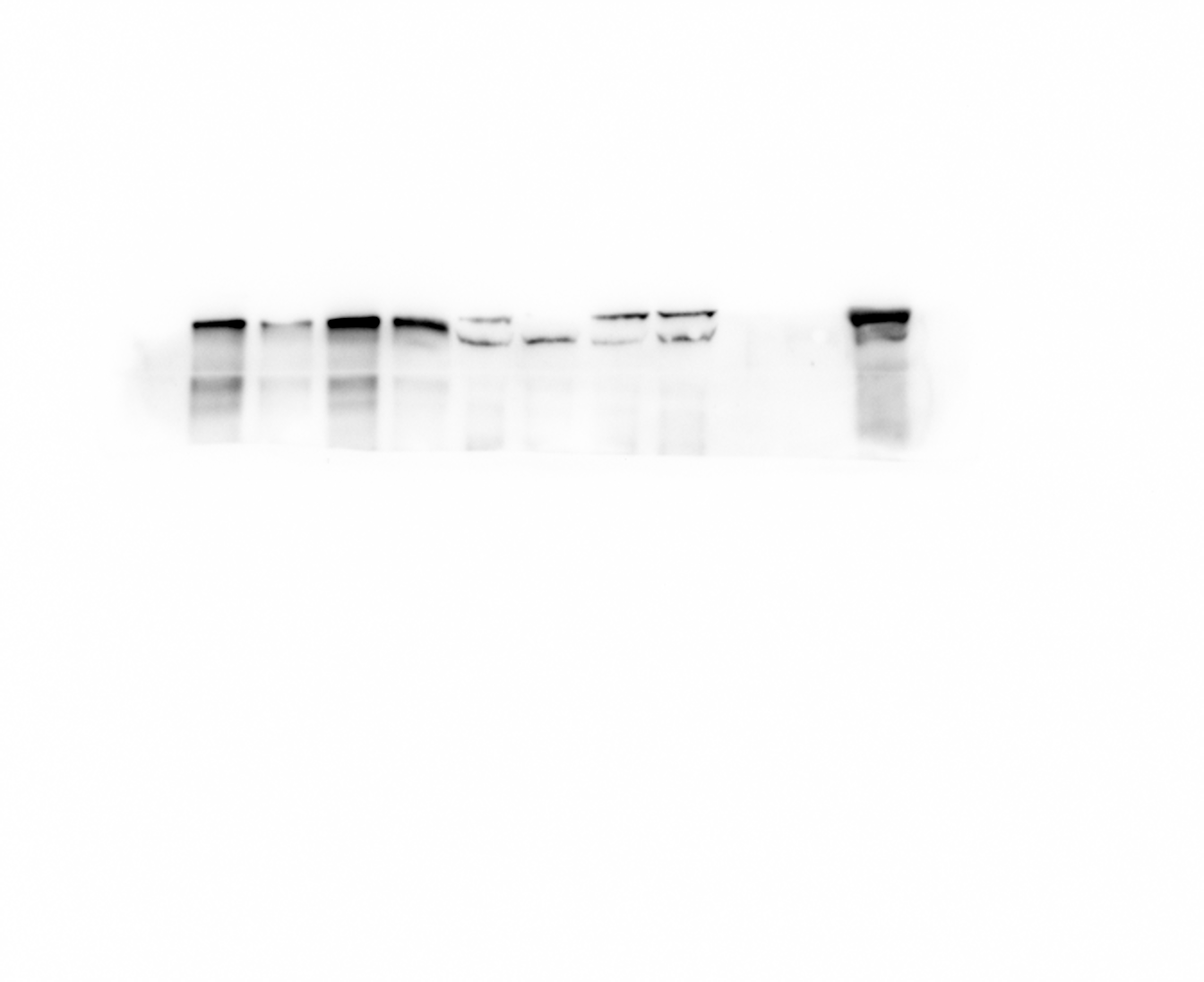


30s

**A549**

**Beas-2b**

**sEV**

**GES**

**SGC7901**

**Cell lysate**

**A549**

**Beas-2b**

**GES**

**SGC7901**

35 kd

CD63

35 kd

CD63

Fig 2 C TSG101


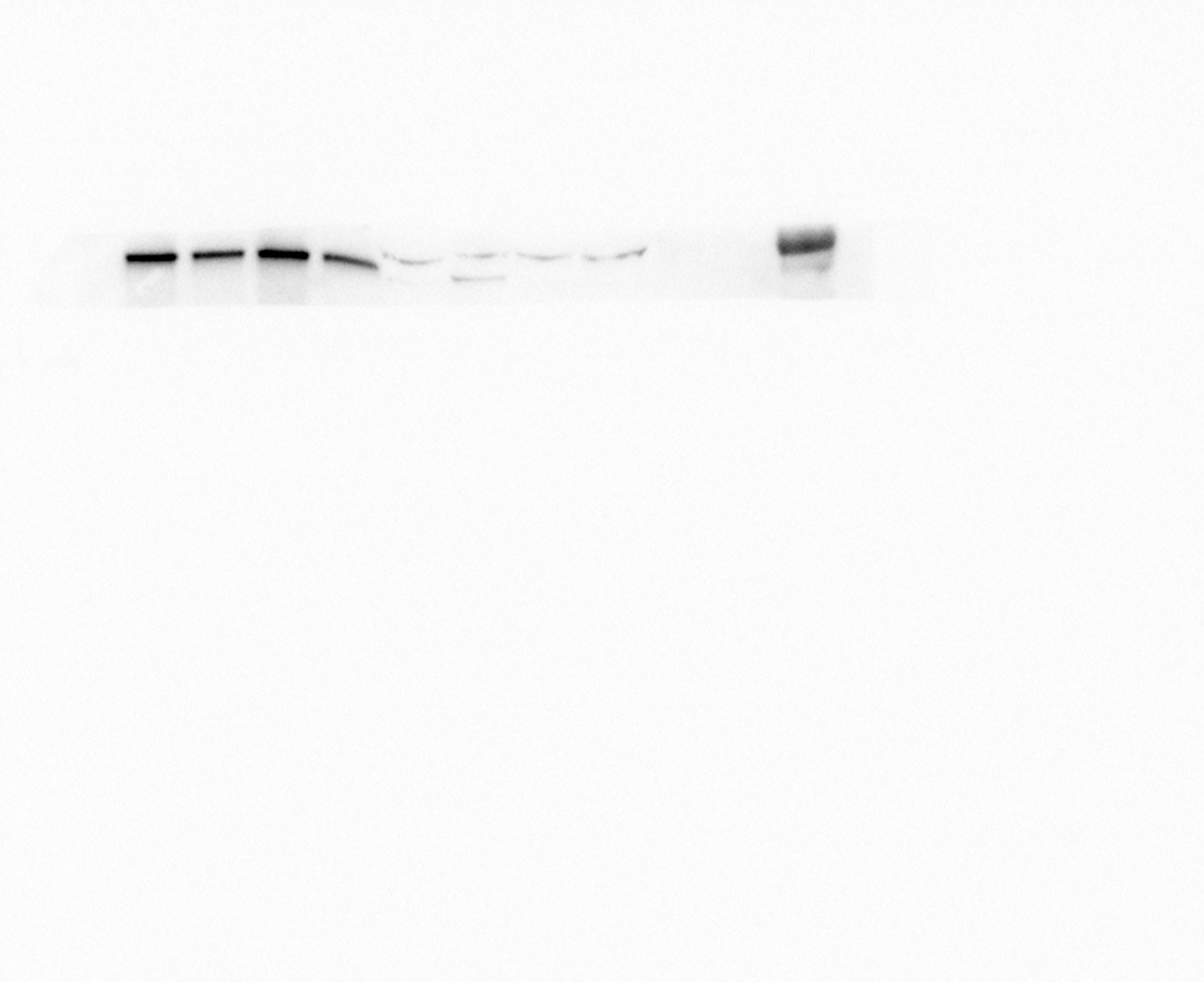


1s

**A549**

**Beas-2b**

**sEV**

**GES**

**SGC7901**

**Cell lysate**

**A549**

**Beas-2b**

**GES**

**SGC7901**

44 kd

TSG101


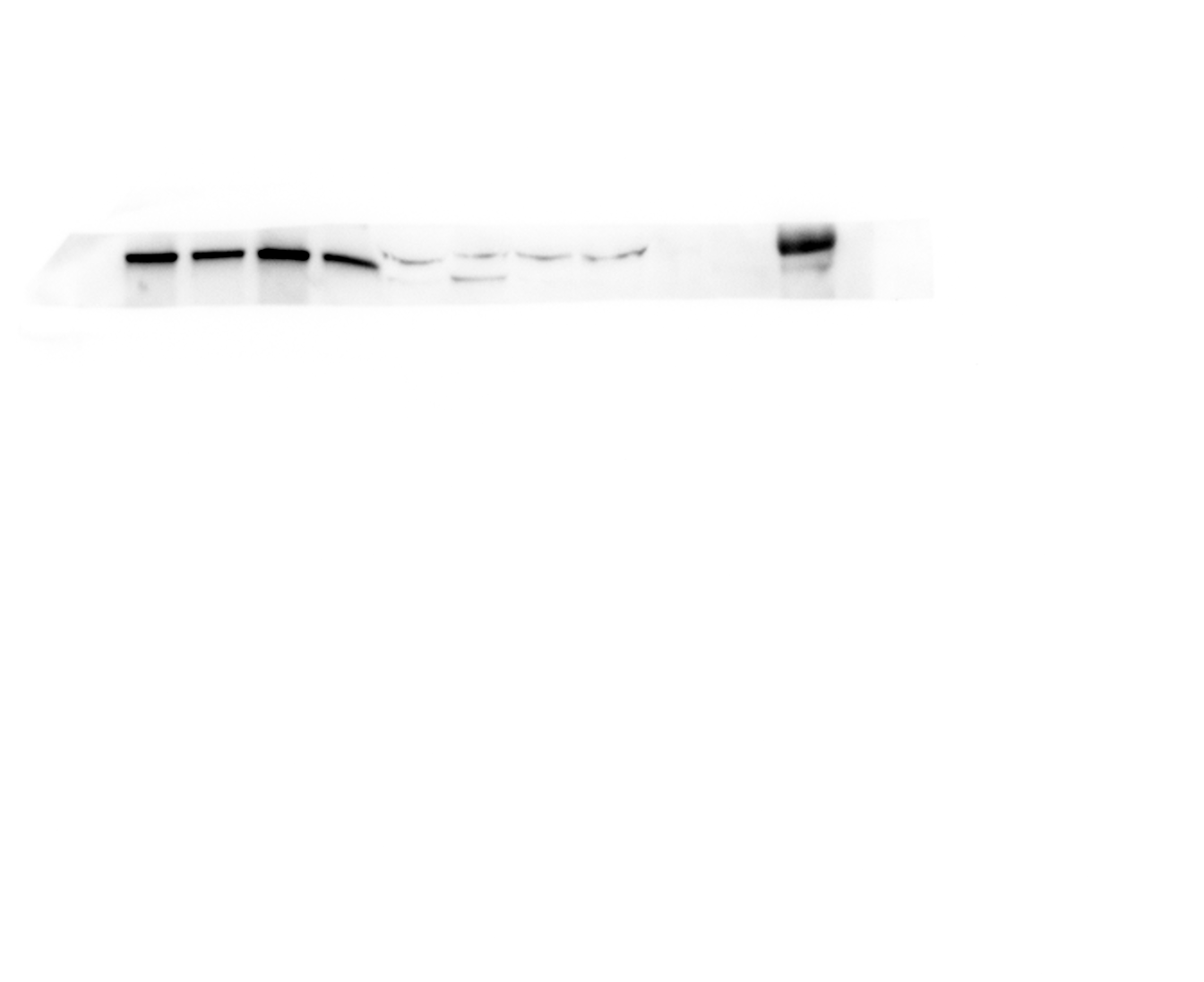


6s

**A549**

**Beas-2b**

**sEV**

**GES**

**SGC7901**

**Cell lysate**

**A549**

**Beas-2b**

**GES**

**SGC7901**

44 kd

TSG101

Fig 2 C Calnexin


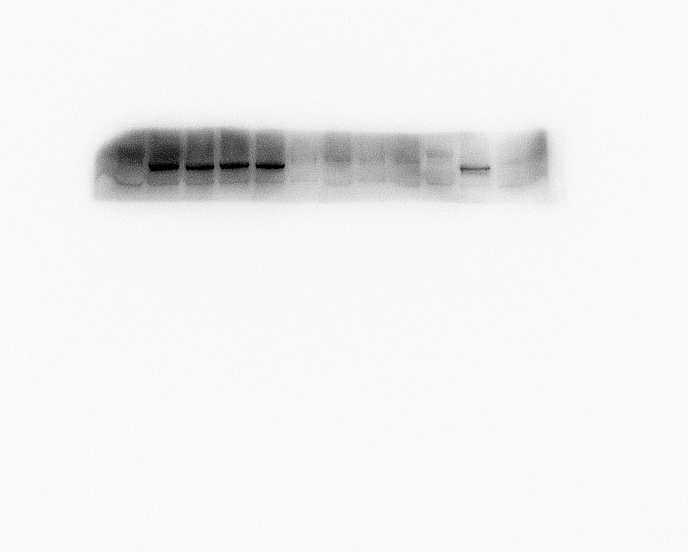


1s

**A549**

**Beas-2b**

**sEV**

**GES**

**SGC7901**

**Cell lysate**

**A549**

**Beas-2b**

**GES**

**SGC7901**

90 kd

Calnexin


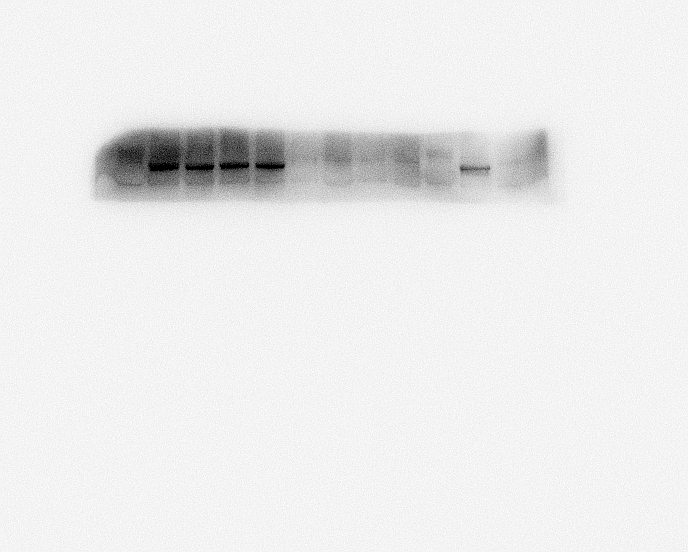


2s

**A549**

**Beas-2b**

**sEV**

**GES**

**SGC7901**

**Cell lysate**

**A549**

**Beas-2b**

**GES**

**SGC7901**

90 kd

Calnexin

Fig 3 B CEA of sEV


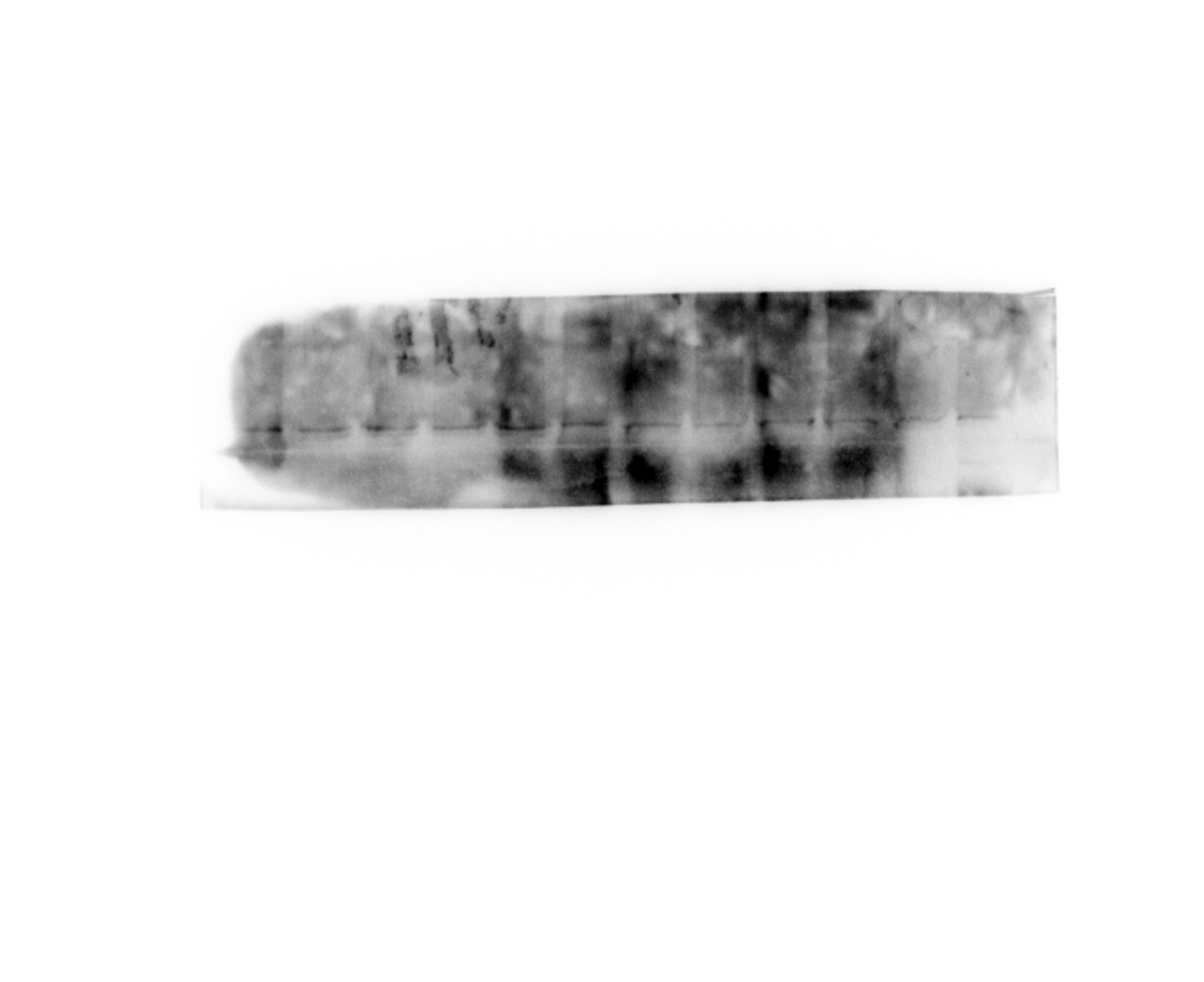


3s

**A549**

**Beas-2b**

**GES**

**SGC7901**

**sEV**

77 kd

CEA


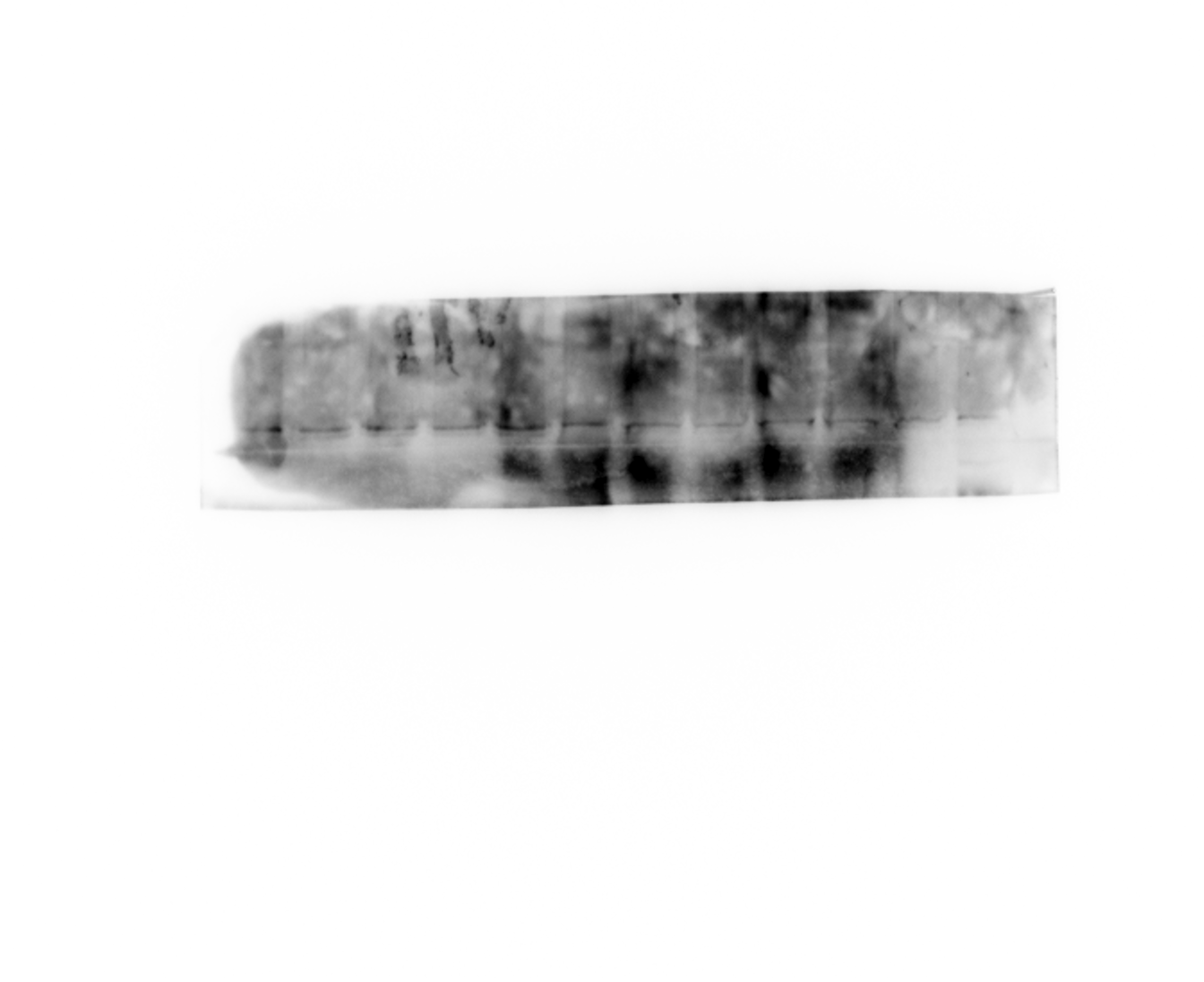


5s

**A549**

**Beas-2b**

**GES**

**SGC7901**

**sEV**

77 kd

CEA

Fig 3 B CEA of Beas-2b and A549 cell lysate


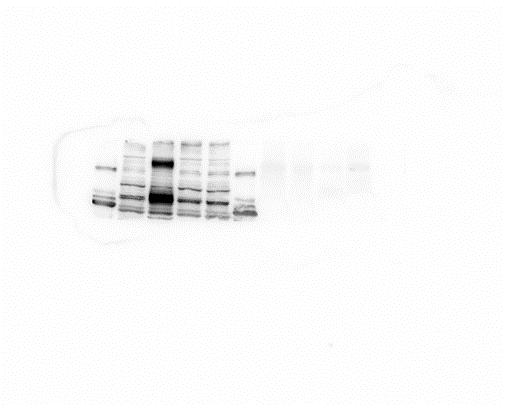


3s

**A549**

**Beas-2b**

Cell lysate

77 kd

CEA


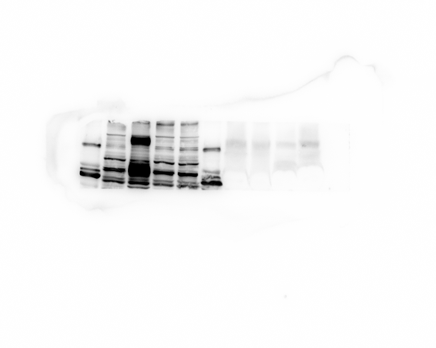


5s

**A549**

**Beas-2b**

Cell lysate

77 kd

CEA

Fig 3 B CEA of GES and SGC7901 cell lysate


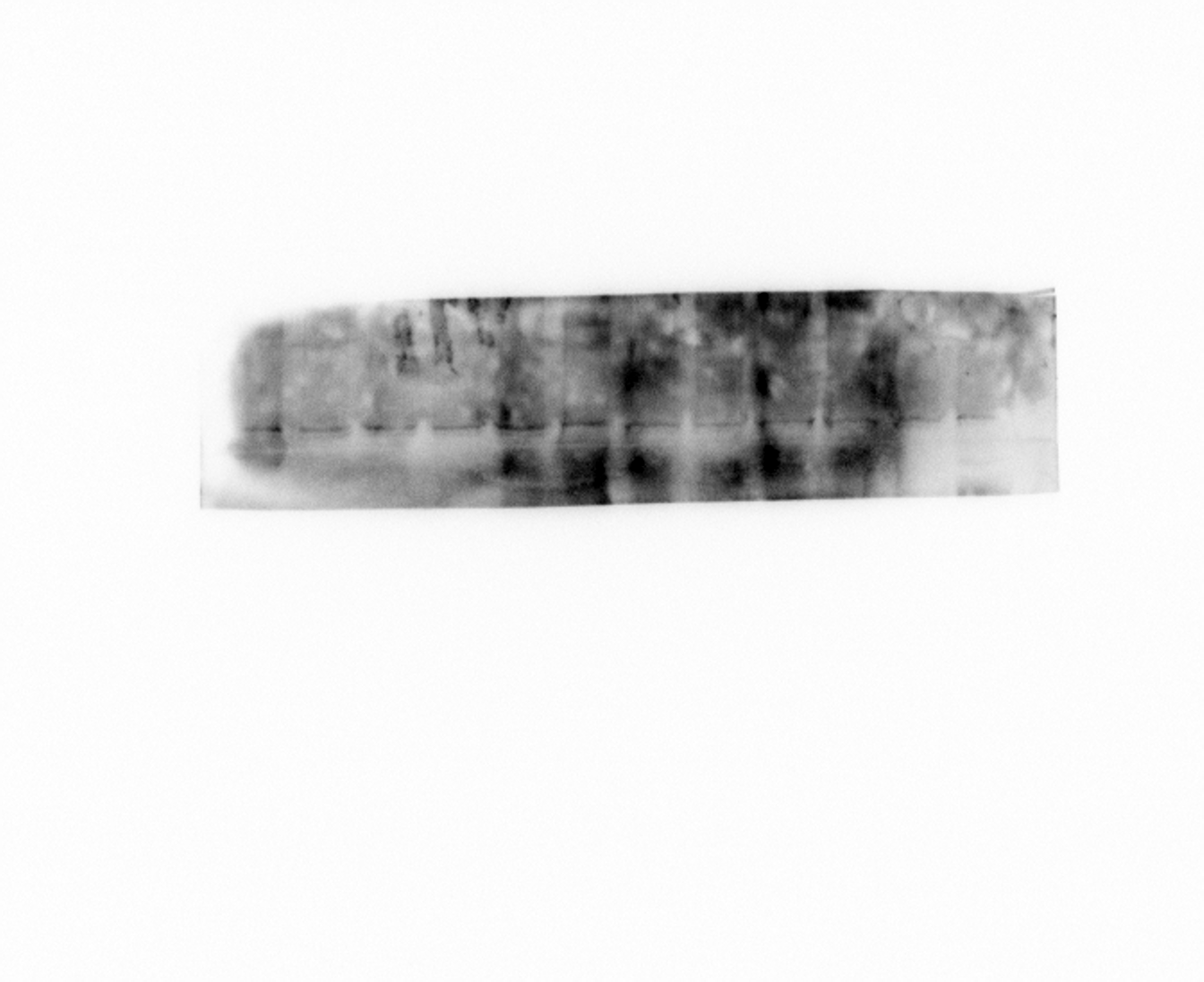


3s

SGC7901

GES

Cell lysate

77 kd

CEA


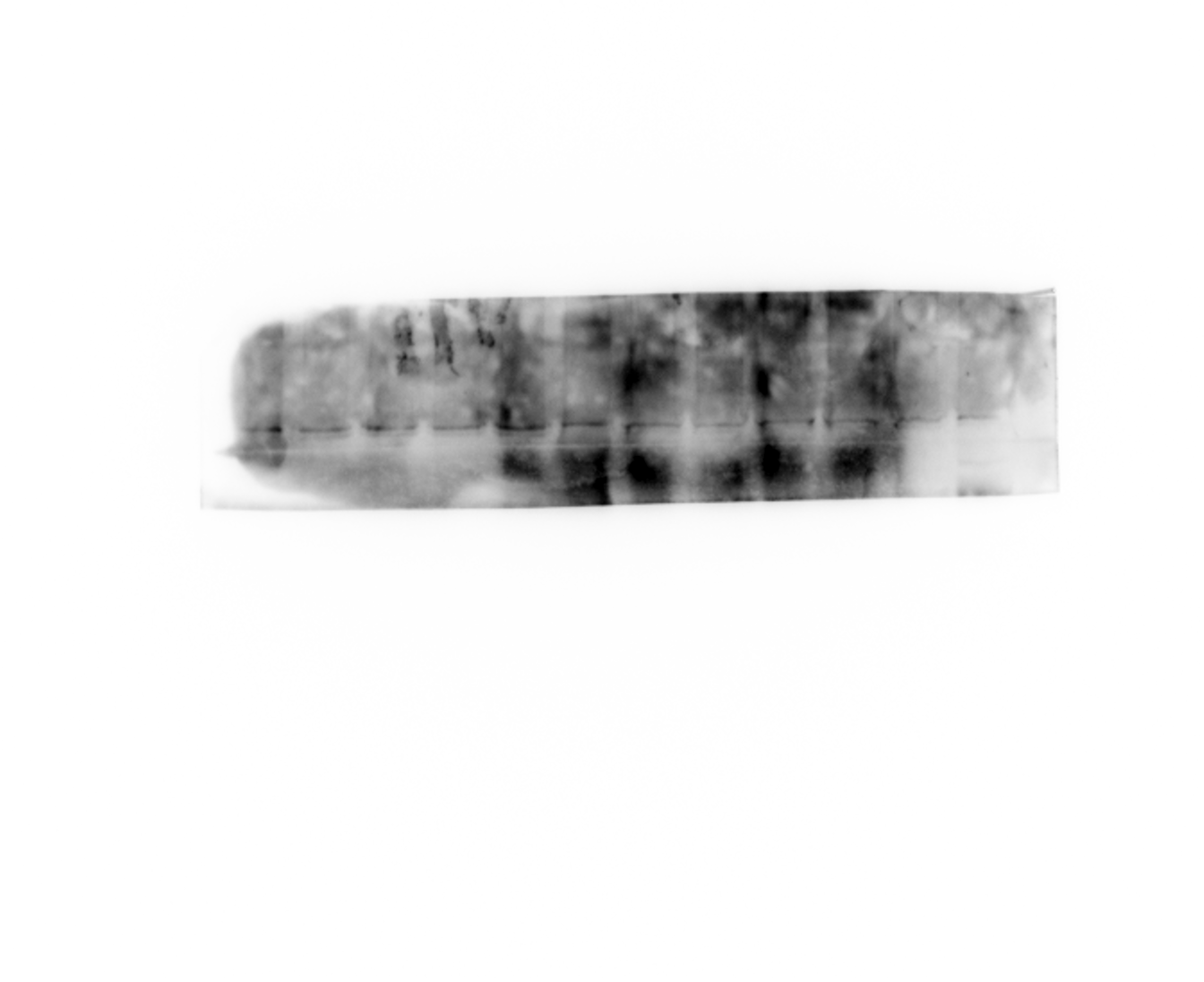


5s

Cell lysate

77 kd

CEA

SGC7901

GES

Fig 3 B GAPDH of Beas-2b and A549 cell lysate


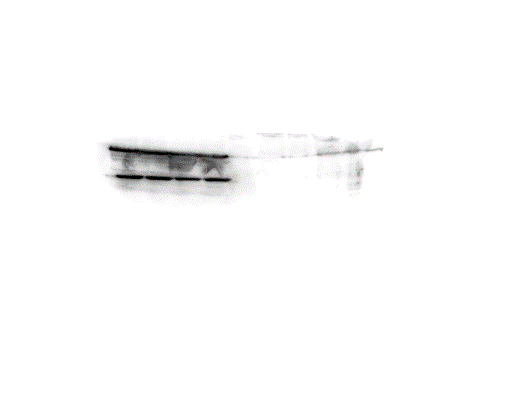


3s

**A549**

**Beas-2b**

Cell lysate

36 kd

GAPDH


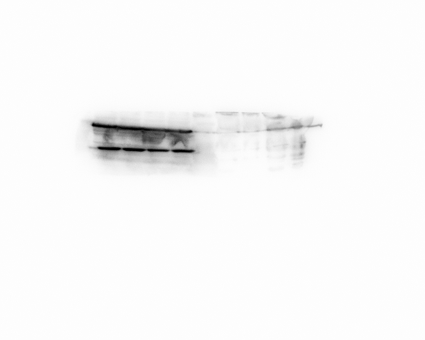


Cell lysate

**A549**

36 kd

GAPDH

4s

**Beas-2b**

Fig 3 B GAPDH of GES and SGC7901 cell lysate


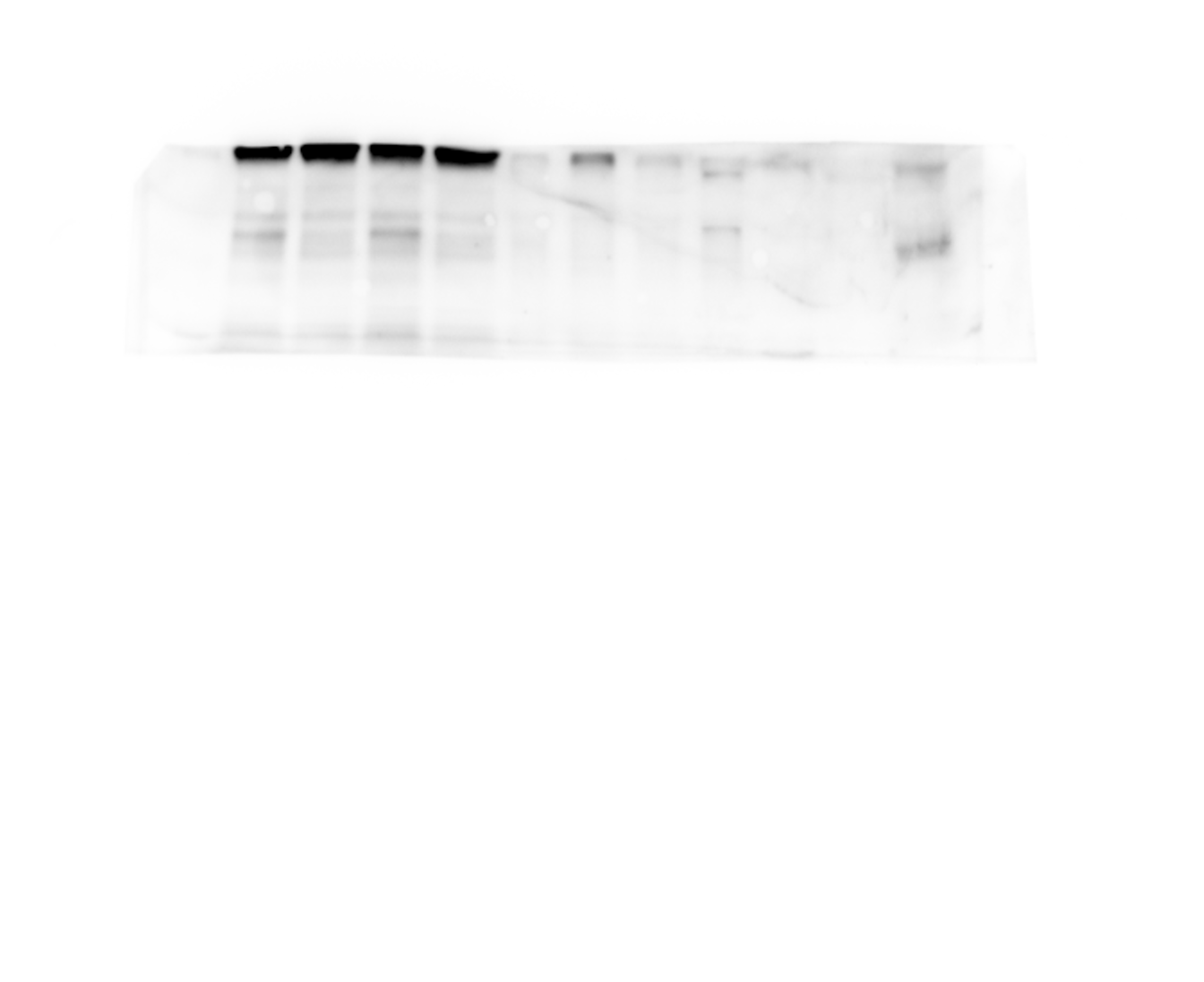


11s

Cell lysate

SGC7901

GES

36 kd

GAPDH


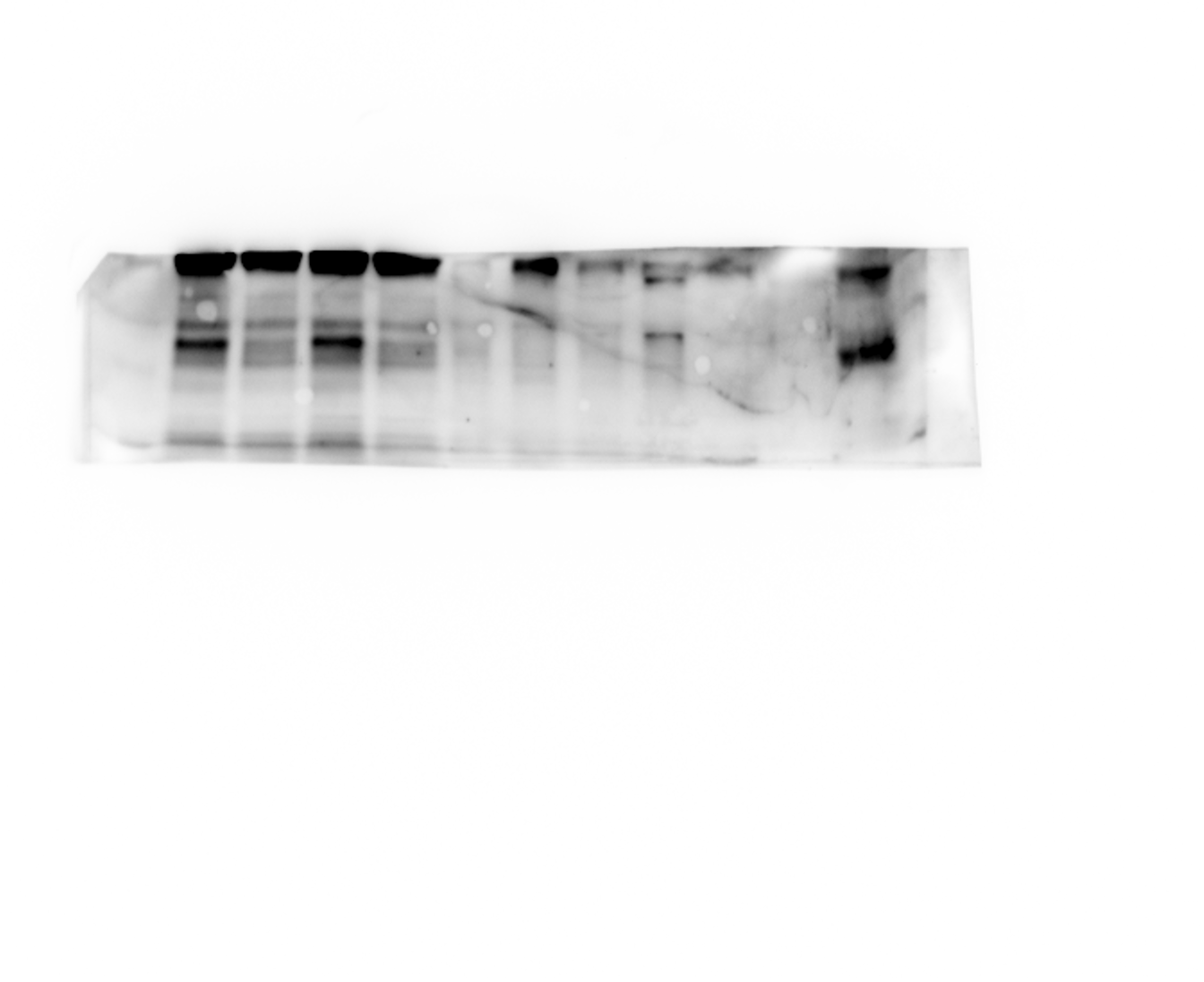


Cell lysate

SGC7901

GES

30s

36 kd

GAPDH
